# Association between insulin resistance and c-reactive protein among Peruvian adults

**DOI:** 10.1186/1758-5996-2-30

**Published:** 2010-05-18

**Authors:** Bizu Gelaye, Luis Revilla, Tania Lopez, Luis Suarez, Sixto E Sanchez, Karin Hevner, Annette L Fitzpatrick, Michelle A Williams

**Affiliations:** 1 University of Washington, Multidisciplinary International Research Training Program, Seattle, WA, USA; 2 Direccion General de Epidemiologia, Ministerio de Salud, Peru; 3 Dos de Mayo Hospital, Lima, Peru; 4 Center for Perinatal Studies, Swedish Medical Center, Seattle Washington, USA

## Abstract

**Objective:**

Insulin resistance (IR), a reduced physiological response of peripheral tissues to the action of insulin, is one of the major causes of type 2 diabetes. We sought to evaluate the relationship between serum C-reactive protein (CRP), a marker of systemic inflammation, and prevalence of IR among Peruvian adults.

**Methods:**

This population based study of 1,525 individuals (569 men and 956 women; mean age 39 years old) was conducted among residents in Lima and Callao, Peru. Fasting plasma glucose, insulin, and CRP concentrations were measured using standard approaches. Insulin resistance was assessed using the homeostasis model (HOMA-IR). Categories of CRP were defined by the following tertiles: <0.81 mg/l, 0.81-2.53 mg/l, and >2.53 mg/l. Logistic regression procedures were employed to estimate odds ratios (OR) and 95% confidence intervals (CI).

**Results:**

Elevated CRP were significantly associated with increased mean fasting insulin and mean HOMA-IR concentrations (p < 0.001). Women with CRP concentration >2.53 mg/l (upper tertile) had a 2.18-fold increased risk of IR (OR = 2.18 95% CI 1.51-3.16) as compared with those in the lowest tertile (<0.81 mg/l). Among men, those in the upper tertile had a 2.54-fold increased risk of IR (OR = 2.54 95% CI 1.54-4.20) as compared with those in the lowest tertile.

**Conclusion:**

Our observations among Peruvians suggest that chronic systemic inflammation, as evidenced by elevated CRP, may be of etiologic importance in insulin resistance and diabetes.

## Background

Insulin resistance (IR), a reduced physiological response of peripheral tissues to the action of insulin, is one of the major causes of type 2 diabetes and plays a critical role in the pathogenesis of cardiovascular diseases (CVDs) [1]. Recent studies have shown that the worldwide prevalence of IR and its associated risk factors have increased markedly [2,3]. IR is believed to be associated with chronic inflammatory response which is characterized by abnormal cytokine production and the activations of pro-inflammatory signaling pathways [4,5].

The systemic inflammatory biomarker C-reactive protein (CRP) when measured in the blood with high sensitivity assay has been reported to be a strong and independent predictor of myocardial infarction, ischemic stroke, type 2 diabetes, and hypertension [6,7]. Several studies have provided strong evidence of association between CRP and CVD risk independent of traditional risk factors, such as cholesterol, blood pressure, alcohol consumption, and smoking habit [7,8]. Accumulating experimental and epidemiologic data now link inflammatory processes to impaired glucose metabolism [9]. It has also been reported that elevated CRP concentrations may reflect not only local inflammation at atherosclerotic lesions but also predict future CVD risk including IR [7,10].

An emerging body of evidence documents associations of elevated CRP concentrations in individuals with IR [10-13]. Few studies, however, have evaluated this association among South Americans. We, therefore, sought to evaluate the relationship between IR and serum CRP among Peruvian adults. Elucidation of the relationship between IR and CRP concentration may lead to an improved recognition of individuals at risk for future diabetes and CVD risk and may help in identification of novel therapeutic targets [9].

## Methods

This study is part of a larger population based study among Peruvian men and women who participated in the Prevalencia de Factores de Riesgo de Enfermedades No-Transmissibles (Prevalence of Risk Factors for Non Transmissible Diseases) referred to as the FRENT study. The primary objective of the FRENT study was to determine the prevalence and risk factors associated to non-transmissible diseases among residents in Lima and Callao, Peru.

Details of the study setting, sampling, and data collection procedures have been described previously [14]. For the study described here, we excluded subjects with diabetes (69), those who were taking lipid lowering drugs (33), and with missing laboratory information (31). The final analyzed sample included 1,525 individuals (569 men and 956 women).

### Data Collection and Variable Specification

Briefly, each participant was interviewed by a trained interviewer using a structured questionnaire validated by the Pan American Health Organization (PAHO) for assessing the prevalence of non transmissible disease (NTD) risk factors [15]. The questionnaire inquired about individuals' age, gender, educational attainment, and medical history via in-person interviews. Height and weight were measured with participants wearing light clothing and no shoes. Waist circumference was measured from the midpoint between the iliac crest and the lower ribs measured at the sides. Hip circumference was measured at the point of the maximum circumference over the buttocks. After participants had been seated for at least 5 minutes, and again after 30 minutes during the interview, a resting blood pressure was measured using a Mercury Desk Sphygmomanometer, these two values were averaged. A follow-up visit to participants' home was made the next morning to collect a blood sample after a 12-hour fast. Aliquots of serum were used to determine participants' fasting glucose concentrations and lipid profiles. Serum triglycerides (TG), total cholesterol, high-density lipoprotein cholesterol (HDL-C), low-density lipoprotein cholesterol (LDL-C), glucose, and insulin were measured at the Peruvian National Institute of Health Laboratory in Lima, Peru. TG concentrations were determined by standardized enzymatic procedures using glycerol phosphate oxidase assay. HDL-C was measured by a chemical precipitation technique using dextran sulfate. Participants' fasting serum glucose (FSG) was determined using standardized glucose oxidase method (glucose oxidase GOD-PAP).

As an index of insulin resistance, we determined the Homeostatis Model Assessment of Insulin Resistance (HOMA-IR) index by dividing the product of fasting glucose (mg/dl) and fasting insulin (μIU/ml) by 405 [16]. The HOMA IR is widely used in clinical trials and has been shown to be correlated with the results obtained from the "gold standard" euglycemic glucose clamp method [1]. Serum CRP concentrations were measured by an ultra-sensitive competitive immunoassay (Dade Behring, Deerfield, Illinois) with the intra- and inter-assay coefficients of variation both < 10% [17]. All laboratory assays were completed without knowledge of participants' medical history.

All subjects provided informed consent and all research protocols were approved by the Institutional Review Boards of National Institute of Health in Peru, Dos de Mayo Hospital, Peru and the Human Subjects Division at the University of Washington, USA.

### Statistical Analyses

We first explored frequency distributions of socio-demographic, clinical and behavioral characteristics of subjects. Continuous variables were expressed as mean ± standard error of means (SEM). Categorical variables were expressed as number (percent, %). Chi-Square tests were used to evaluate differences in the distribution of categorical variables. Student's t tests were used to evaluate differences in mean distributions. Associations of CRP with HOMA-IR and other continuous covariates (e.g., insulin and glucose) were determined using the Pearson's correlation coefficients. The distribution of fasting insulin, fasting glucose, and HOMA-IR were skewed to the right side, and the values were transformed to the natural logarithms in the analysis. Thus, the presented means were always geometric means. Mean values of fasting insulin, fasting glucose, and HOMA-IR were compared across the different concentrations of CRP using linear regression procedures. Categories of CRP were defined by the following tertiles: <0.81 mg/l, 0.81-2.53 mg/l, and >2.53 mg/l. The group with the lowest tertile of CRP (<0.81) served as the reference group. Confounding variables were considered *a priori *on the basis of their hypothesized relationship with IR and CRP. Multiple linear regression procedures were used to adjust for covariates of interest: age, BMI, and current smoking status. Presence of IR (yes/no) was defined as being in the highest quartile of HOMA-IR (>5.12). Logistic regression procedures were employed to estimate odds ratios (OR) and confidence intervals (CI) of IR according to tertiles of CRP. In multivariate analysis, tests for linear trend across increasing categories of CRP were conducted by treating the three-level CRP-concentration variable as an ordinal variable. Since previous studies have noted that the effect of CRP on IR differs among men and women, regression analyses were reported for men and women separately. All statistical analyses were performed using SPSS (version 14.0, SPSS Inc. Chicago, IL, USA) software. All reported p-values are two tailed and confidence intervals were calculated at the 95% concentration.

## Results

Characteristics of study participants according to gender are shown in Table 1. The percentages of current smokers, mean value of waist circumference, triglycerides, systolic and diastolic blood pressure were significantly higher among men than women. Women had significantly higher mean age, high density lipoprotein-cholesterol, total cholesterol, and low density lipoprotein-cholesterol. There was no significant gender difference in the values of CRP, fasting insulin and fasting glucose. Values of CRP were positively associated with fasting insulin and HOMA-IR in men (*r *= 0.190 and 0.166 respectively <0.001) and women (*r *= 0.157 and 0.153 respectively, p < 0.001).

**Table 1:** Characteristics of the Study Population, Lima, Peru 2006.

Characteristics	Women (N = 956)	Men (N = 569)	**P-Value
Age	39.9 ± 14.5	38.3 ± 15.9	
Ever smoking (%)	16.0	44.5	<0.001
Current smoking (%)	9.6	32.0	<0.001
BMI (kg/m^2^)	26.6 ± 4.8	26.1 ± 4.0	0.042
Waist Circumference (cm)	88.3 ± 11.5	92.0 ± 10.6	<0.001
Hip circumference (cm)	99.6 ± 10.7	98.8 ± 8.4	0.111
Total cholesterol (mg/dl)	186.0 ± 42.0	179.6 ± 39.1	0.003
HDL cholesterol (mg/dl)	48.5 ± 0.4	42.6 ± 0.4	<0.001
LDL cholesterol (mg/dl)^†^	114 (94-138)	110 (92-135)	0.042
Triglycerides (mg/dl)^†^	104(69.0-156.0)	124(84.0-188.5)	<0.001
Systolic blood pressure (mm Hg)	113.4 ± 16.1	119.9 ± 13.8	<0.001
Diastolic blood pressure (mm Hg)	71.5 ± 10.4	75.5 ± 10.6	<0.001
CRP (mg/l)^†^	1.5 (0.6-3.4)	1.5 (0.6-3.8)	0.252
Fasting insulin(μU/ml)^†^	15.0 (10.2-22.8)	16.0 (10.6-23.9)	0.319
Fasting glucose(mmol/l)	4.8 ± 1.0	4.9 ± 1.1	0.180
HOMA-IR^†^	3.1 (2.1-4.9)	3.4 (2.2-5.3)	0.167

* Data are mean ± SD, percentages or median (interquartile range) for variables with skewed distributions ^†^Test of significance was based on log-transformed values

** P-value from Chi-Square test for categorical variables or from Student's t-test for continuous variables

Table 2 shows the adjusted (age, BMI, current smoking status) mean values of fasting insulin, fasting glucose, and HOMA-IR according to categories of serum CRP and gender. Elevated CRP concentrations were significantly associated with increased fasting insulin and HOMA-IR concentrations (trend test p < 0.001). Among women, there was no observed difference in mean fasting glucose whereas men had significantly lower fasting glucose with increased CRP concentrations (trend test p = 0.005).

**Table 2:** *Adjusted Geometric Mean Values of Fasting Insulin, Fasting Glucose, and HOMA-IR, According to C-Reactive Protein Concentration and Gender.

C-Reactive Protein (mg/l)	n	Geometric Mean of fasting insulin (μU/ml) (95% CI)	Geometric Mean of fasting glucose (mmol/l) (95% CI)	Geometric Mean of HOMA-IR^†^(95% CI)
**Women**				
<0.81	325	2.63 (2.56-2.69)	1.56 (1.55-1.58)	1.08 (1.01-1.15)
0.81-2.53	320	2.76 (2.72-2.81)	1.56 (1.55-1.57)	1.21 (1.17-1.26)
>2.53	310	2.90 (2.83-2.97)	1.56 (1.54-1.57)	1.35 (1.27-1.43)
***Trend p-value***		*<0.001*	*0.593*	*<0.001*
**Men**				
<0.81	183	2.59 (2.50-2.68)	1.60 (1.58-1.62)	1.08 (0.99-1.17)
0.81-2.53	189	2.77 (2.71-2.82)	1.58 (1.56-1.59)	1.23 (1.17-1.29)
>2.53	197	2.94 (2.85-3.02)	1.55 (1.54-1.58)	1.38(1.29-1.47)
***Trend p-value***		*<0.001*	*0.005*	*<0.001*

^† ^HOMA-IR-Homeostasis model assessment of insulin resistance.

*Mean values were adjusted for age, current cigarette smoking status, and body mass index. Separate models were estimated for women and men.

Serum CRP concentrations were statistically significantly and positively associated with prevalent IR in both women and men in our population (trend test p-values < 0.001) (Table 3). After adjusting for age, smoking status and body mass index the ORs and 95% confidence intervals across increasing tertiles for women were:, 1.35 (95% CI 0.91-1.99), and 2.18 (95% CI 1.50-3.17). The corresponding ORs and 95% confidence intervals for men we as follows: 2.67 (95% CI 1.61-4.44), and 2.58 (95% CI 1.55-4.28).

**Table 3:** Adjusted Odds Ratios (OR) and 95% Confidence Intervals (95% CI) of Insulin Resistance among Peruvian Women and Men According to Tertiles of Serum CRP Concentrations.

	Insulin Resistance		
		
C-Reactive Protein (mg/l)	No n (%)	Yes n (%)	*Adjusted Odds Ratio (95% CI)
**All**			
<0.81	422 (36.9)	86 (22.6)	1.00 (Reference)
0.81-2.53	376 (32.9)	133 (34.9)	1.75 (1.28-2.35)
>2.53	346 (30.2)	162 (42.5)	2.29 (1.70-3.09)
	***Trend p-value***		<0.001
**Women**			
<0.81	267 (36.7)	58 (25.3)	1.00 (Reference)
0.81-2.53	249 (34.3)	71 (31.0)	1.35 (0.91-1.99)
>2.53	211 (29.0)	100 (43.7)	2.18 (1.50-3.17)
	***Trend p-value***		<0.001
**Men**			
<0.81	155 (37.2)	28 (18.4)	1.00 (Reference)
0.81-2.53	127 (30.5)	62 (40.8)	2.67 (1.61-4.44)
>2.53	135 (32.4)	62 (40.8)	2.58 (1.55-4.28)
	***Trend p-value***		0.001

*Adjusted for age, current cigarette smoking status, and body mass index. Separate models were estimated for women and men.

## Discussion

The present study has shown that IR was associated with CRP, a marker of systemic inflammation, in the Peruvian adult population. After adjusting for BMI, age, and current smoking status, those in the upper tertile (CRP concentration >2.53 mg/l) had more than 2-fold increased risk of IR, compared with those in lowest tertile (CRP concentration <0.81 mg/l) in both men and women. Our observations among Peruvians are in agreement with reports from cross sectional and prospective studies of other populations that showed associations between IR and CRP [10-13]. These associations are explained by different possible mechanisms.

A number of studies have reported that low-grade inflammation is a novel risk factor in all stages of atherosclerosis and acute coronary syndrome [18,19]. It appears that adipose tissue in general; visceral adipose tissue in particular, plays a key role in regulating inflammation [10]. Notably, CRP is primary synthesized in the liver and regulated by the pro-inflammatory cytokine IL-6 and tumor necrosis factor-alpha (TNF-α) [20] in adiposities. This in part suggests that the associations of CRP concentrations with fasting insulin, fasting glucose, and HOMA-IR could be due to the presence of a chronic systemic sub-clinical inflammation [10].

It has also been shown that CRP, in addition to providing downstream integration to overall cytokine activation, is bound to the membranes of damaged vascular cells where it activates complement proteins or enhances the production of thrombogenic agents [21,22] depending on conventional risk factors and other markers of inflammation [23]. These instances of vascular inflammation may contribute to the development of insulin resistance [21,22] although the mechanisms are not fully known [23].

Some important study limitations must be considered when interpreting the results of our study. First, because of this cross-sectional data collection design, we cannot be certain of the temporal relation between elevated CRP concentration and risk of insulin resistance. Inferences concerning the risk for incident insulin resistance and type 2 diabetes, however, will be enhanced with data from prospective studies among Peruvians. Second, a single measurement of serum CRP is not likely to provide a time-integrated measure of inflammation status. However, several investigators have noted that CRP concentrations are stable over a long period of time [7,24]. Third, direct measures of central adiposity were not used. Studies have shown that among middle-aged adults, BMI is strongly correlated with more invasive direct measures of central adiposity [25,26]. Fourth, HOMA-IR was used for assessing insulin resistance in our population-based epidemiological study. The euglycemic glucose clamp has been reported to be the gold standard method for evaluating insulin resistance [16]. Because of its invasiveness, complexity and expense, the euglycemic glucose clamp method is of limited use for clinical screening exams and population-based epidemiological studies. The results from HOMA-IR have been correlated with the results obtained from the euglycemic hyperinsulinemic clamp technique [1,16].

Our understanding of CRP over the last decade has increased markedly. CRP has been shown to be a useful marker and mediator of inflammation and a powerful predictor of future CVD event [9]. This increased recognition has led for the Centers for Disease Control and Prevention and the American Heart Association to publish guidelines that endorse the use of CRP as the only inflammatory biomarker currently available with adequate standardization and predictive value that is suitable as an adjunct to traditional risk factor screening [27]. Collectively, available evidence suggests that systemic inflammation, as evidenced by elevated CRP, may be of etiologic importance in insulin resistance and diabetes. Our observation among Peruvians raise the possibility that inflammatory markers, like CRP, might provide an adjunctive method for early detection of type 2 diabetes although further investigation is needed to confirm this observation.

## Competing interests

The authors declare that they have no competing interests.

## Authors' contributions

LR, TL, LS, and SES participated in the design of the study and carried out data collection. BG and MAW participated in statistical analysis, interpretation, and drafted the manuscript. KH participated in the laboratory work. AF, SES, and LR participated in interpretation of results and provided critical review of the manuscript. All authors read and approved the final manuscript
